# Minieditorial: Risco de FA após Ablação de Flutter Dependente de Istmo Cavo-Tricuspídeo: Vale a Pena Fazer a Ablação da FA Simultaneamente?

**DOI:** 10.36660/abc.20200316

**Published:** 2020-05-22

**Authors:** Alvaro Valentim Lima Sarabanda

**Affiliations:** 1 Instituto de Cardiologia do Distrito Federal BrasíliaDF Brasil Instituto de Cardiologia do Distrito Federal, Brasília, DF – Brasil; 2 Fundação Universitária de Cardiologia BrasíliaDF Brasil Fundação Universitária de Cardiologia (FUC), Brasília, DF – Brasil

**Keywords:** Arritmias Cardíacas, Flutter Atrial, Condução, Ablação por Radiofrequência, Istmo-Cavo Tricuspídeo, Fibrilação Atrial/prevenção e controle

O *flutter* atrial (FLA) dependente do istmo cavotricuspídeo (FLA-ICT) é uma arritmia cardíaca frequente que cursa com sintomas limitantes e tem associação ao risco aumentado de acidente vascular cerebral (AVC) e desenvolvimento ou agravamento de insuficiência cardíaca. O substrato anatômico/eletrofisiológico do FLA-ICT é uma combinação de condução lenta pelo istmo de tecido atrial entre o anel tricúspide e a veia cava inferior, associada ao bloqueio funcional da condução ao longo da crista terminal e da crista (*ridge*) de Eustáquio, permitindo o surgimento e a perpetuação de um circuito macrorreentrante no átrio direito.^1,2^

Pelo fato de o substrato anatômico/eletrofisiológico do FLA-ICT ser bem-definido e os resultados do tratamento com fármacos antiarrítmicos serem insatisfatórios, a ablação por radiofrequência do FLA, por meio da criação de uma lesão linear entre o anel tricúspide e a veia cava inferior (istmo cavotricuspídeo), tem sido preconizada como uma terapia segura e eficaz, com baixo risco de complicações (1% ou menos) e índices de sucesso acima de 90%.^1,2^

Embora a ablação por radiofrequência do FLA-ICT apresente elevadas taxas de sucesso, um contingente significativo de pacientes submetidos ao procedimento apresentará um primeiro episódio de fibrilação atrial (FA) no decorrer do período de seguimento clínico.^1,3-5^ Diversas evidências têm sugerido que a ocorrência de novo episódio de FA não seja devido à pró-arritmia resultante da ablação do ICT, retratando, no entanto, outra face da mesma atriopatia, a qual predispõe os pacientes aos dois tipos de arritmias.^6^ Assim, a ablação do ICT não é capaz de impedir a ocorrência de novo episódio de FA. Esse aspecto foi relatado em diversos outros estudos que avaliaram a ocorrência de novas arritmias atriais, incluindo FA, após a ablação do FLA-ICT.^1,3-5^

Nesta edição dos *Arquivos Brasileiros de Cardiologia*, Bianco et al.,^7^ avaliaram a incidência e os preditores de ocorrência de primeiro episódio de FA após ablação do FLA-ICT em série de 84 pacientes sem registro prévio de FA, tendo sido os dados analisados retrospectivamente. Durante o procedimento, houve uma única complicação (1,2%): a embolização da ponta de uma bainha longa utilizada para estabilizar o cateter de ablação, a qual foi removida sem necessidade de intervenção cirúrgica. Durante o seguimento médio de 26±18 meses, 10 (11,5%) pacientes apresentaram recorrência de FLA e 45 (53,6%) apresentaram novo episódio de FA. No entanto, não foram identificadas variáveis preditoras de ocorrência de FA no seguimento clínico.^7^

O estudo de Bianco et al.,^7^ está em concordância com estudos prévios de pacientes submetidos à ablação do FLA-ICT.^1,3-5^ Todavia, a questão colocada em evidência pelos autores é se devemos realizar a ablação da FA e do FLA-ICT simultaneamente nos pacientes com FLA sem histórico de FA. Como ressaltado por Bianco et al.,^7^ o aparecimento de FA após ablação do FLA-ICT apresenta grande relevância clínica devido ao elevado risco de eventos tromboembólicos associados à FA, em especial o AVC. A presença de FA está associada a um aumento de quatro a cinco vezes no risco de AVC isquêmico. Nesse sentido, em estudo incluindo população de pacientes submetidos à ablação do FLA-ICT, a incidência de AVC ao longo de seguimento médio de 40 meses foi cerca de 4 vezes maior do que na população geral.^8^ Por esse motivo, tendo em vista a elevada incidência de FA nessa população, a interrupção do uso de anticoagulantes orais pode expô-la ao risco aumentado de eventos tromboembólicos, devendo ser avaliada a manutenção da anticoagulação oral após ablação do FLA-ICT, levando-se em conta o escore CHA2DS2-VASC do paciente, de forma semelhante aos pacientes com FA.^2,9^

Adicionalmente, um contingente significativo de pacientes continua sintomático em decorrência da FA que se manifesta após ablação do FLA-ICT. Um segundo procedimento de ablação pode ser, então, necessário para o controle da FA. Apesar de o isolamento elétrico de veias pulmonares (Isol-VPs), necessário para o tratamento da FA, ser um procedimento de maior complexidade, envolvendo a realização de punções transeptais, ablação atrial mais extensa e necessidade de utilização de equipamento de mapeamento tridimensional, com maiores riscos e custos comparativamente à ablação de FLA-ICT, uma alternativa a ser considerada é a realização combinada de um único procedimento de ablação para eliminar ambas as arritmias, evitando-se uma segunda intervenção, questão colocada em evidência pelo estudo de Bianco et al.^7^

Nesse contexto, evidências recentes sugerem que a ablação combinada do ICT com o Isol-VPs pode ser uma estratégia eficaz para a prevenção de ocorrência de FA em pacientes com FLA-ICT e sem história prévia de FA.^10-12^ O estudo PREVENT AF I incluiu 50 pacientes com FLA-ICT e sem histórico de FA, randomizados 1:1 entre a estratégia de ablação do ICT isoladamente *versus* ablação do ICT associado ao Isol-VPs com crioablação (ICT + Isol-VPs).^10^ Nova FA foi registrada em 52% dos pacientes submetidos à ablação isolada do ICT versus 12% no grupo ICT + Isol-VPs (p = 0,003), no seguimento de 1 ano. Posteriormente, Romanov et al.,^11^ reportaram o seguimento estendido (3 anos) do estudo PREVENT AF I, observando uma taxa livre de taquiarritmia atrial/FA significativamente maior no grupo de ICT + Isol-VPs, em comparação ao grupo de ablação isolada do ICT (48% *vs.* 20%, P = 0,01). É importante ressaltar que não houve eventos adversos no grupo ICT + Isol-VPs, mas ocorreram 2 episódios de AVC no grupo de ablação isolada do ICT. A análise multivariada identificou o sexo masculino e a idade > 55 anos como fatores preditores de ocorrência de arritmia atrial no seguimento. Por fim, o estudo REDUCE AF^12^ randomizou 216 pacientes com FLA-ICT para ablação isolada do ICT *versus* ablação combinada do ICT + Isol-VPs, observando uma redução dos episódios de FA com a estratégia de ablação combinada, mas às custas de aumento do tempo de duração do procedimento e do uso da fluoroscopia. Na análise *post hoc*, o benefício da estratégia de ablação combinada foi limitado aos pacientes com > 55 anos de idade, em concordância com os resultados de Romanov et al.,^11^

Recentemente, Gula et al.,^13^ realizaram uma análise de custo-efetividade, comparando a estratégia de ablação combinada do ICT + Isol-VPs *versus* a estratégia de ablação sequencial, ou seja, com a possibilidade de realização dos 2 procedimentos separadamente caso houvesse o aparecimento de FA. Por meio de projeções baseadas em estudos que reportaram as taxas de aparecimento de nova FA após ablação do FLA-ICT, as taxas de sucesso do Isol-VPs, bem como os riscos e custos dos procedimentos de ablação do FLA-ICT e do Isol-VP, os autores propuseram que a estratégia de ablação combinada do ICT + Isol-VPs não apresenta, via de regra, benefícios do ponto de vista financeiro e dos riscos envolvidos no procedimento. Deve-se ponderar, no entanto, as limitações desse estudo e considerar que a estratégia combinada de ablação do ICT + Isol-VPs poderia demonstrar um benefício favorável se fosse aplicada somente aos pacientes identificados com maior risco de apresentarem FA no seguimento clínico.

Nesse contexto, uma limitação significativa do estudo de Bianco et al.,^7^ foi o tamanho restrito da população estudada, o que pode ter delimitado a identificação de preditores para a ocorrência de FA após a ablação do FLA-ICT. Como reconhecido pelos autores,^7^ caso fosse possível identificar um perfil de risco para a ocorrência de FA após a ablação ICT, uma abordagem combinada, incluindo a ablação das duas arritmias, poderia ser indicada seletivamente nos pacientes com maior risco de desenvolver FA.

Em resumo, o estudo de Bianco et al.,^7^ fornece evidências adicionais de que a ablação do ICT é um procedimento seguro e eficaz, mas soluciona apenas parcialmente os problemas clínicos dos portadores de FLA-ICT isoladamente, uma vez que a ocorrência de FA após ablação do ICT é frequentemente observada. Todavia, com relação à questão colocada em evidência pelos autores,^7^ ainda não há dados suficientes para a indicação de ablação combinada no tratamento do FLA-ICT visando à prevenção da ocorrência de FA. Estudos prospectivos, com maior número de pacientes e seguimento mais longo, serão necessários para avaliar os benefícios de uma ablação simultânea.
