# Supplementary material for: Minieditorial: Risco de FA após Ablação de Flutter Dependente de Istmo Cavo-Tricuspídeo: Vale a Pena Fazer a Ablação da FA Simultaneamente?
Source: Arq Bras Cardiol. 2020 May 22;114(5):783–5. [Article in Portuguese] doi: 10.36660/abc.20200316 (PMC8387005; doi:10.36660/abc.20200316)
Supplement: Translation

# Translation: Short Editorial: Risk of Atrial Fibrillation after Ablation of Cavotricuspid Isthmus-Dependent Atrial Flutter: Is Combined Ablation of Atrial Fibrillation Worthwhile?

**Authors:** Alvaro Valentim Lima Sarabanda

**Affiliations:** 1 Brazil Instituto de Cardiologia do Distrito Federal, Brasília, DF – Brazil; 2 Brazil Fundação Universitária de Cardiologia (FUC), Brasília, DF – Brazil

**Keywords:** Arrhythmias, Cardiac, Atrial Flutter, Conduction, Radiofrequency Ablation, Isthmus Cavo-Tricuspid, Atrial Fibrillation/prevention and control

Cavotricuspid isthmus (CTI)-dependent atrial flutter (AFL) is a common cardiac arrhythmia that can cause significant symptoms and is associated with an increased risk of stroke and the development or worsening of heart failure. The anatomic/electrophysiological substrate underlying AFL is a combination of slow conduction in the isthmus of atrial tissue between the tricuspid annulus and the inferior vena cava and conduction block along the crista terminalis and Eustachian ridge, enabling the emergence and perpetuation of a macro-reentrant circuit in the right atrium.^1,2^

Because of the well-defined anatomic/electrophysiological substrate and the unsatisfactory results of antiarrhythmic drug therapy in treating AFL, radiofrequency catheter ablation, by means of creating a linear lesion from the tricuspid annulus to the inferior vena cava (cavotricuspid isthmus) under fluoroscopic and electrocardiographic guidance, is an established interventional procedure, with a low risk of complications (1% or less) and success rates over 90%.^1,2^

Although acutely highly successful, a significant number of patients with successful (CTI)-dependent AFL ablation will develop atrial fibrillation (AF) during the follow-up period.^1,3-5^ Rather than proarrhythmia, it has been suggested that the occurrence of new AF reflects manifestation of the same atrial disease that predisposes patients to both arrhythmias.^6^ Thus, elimination of the CTI-dependent AFL circuit does not prevent new AF. This effect has been reported in multiple studies examining the emergence of atrial arrhythmias, including AF, following CTI ablation for AFL.^1,3-5^

In this issue of the *Arquivos Brasileiros de Cardiologia*, Bianco et al.^7^ explore the incidence and predictors of AF following ablation of AFL. They present a series of 84 patients without any prior history of AF undergoing catheter ablation of CTI-dependent AFL, with data analyzed retrospectively. There was only one periprocedural complication (1.2%), an embolization of the tip of the long sheath used for stabilization of the 8mm tip ablation catheter, which was successfully removed without surgical intervention. During a mean follow-up of 26±18 months, 10 (11.5%) patients had recurrence of AFL and 45 (53.6%) had a first episode of AF. However, no predictive variables for the occurrence of AF were identified in the clinical follow-up.^7^

The study of Bianco et al.^7^ is in agreement with previous studies of patients undergoing CTI-dependent AFL ablation.^1,3-5^ However, the question raised by the authors is whether we should perform a concomitant AF ablation in patients undergoing CTI-dependent AFL ablation without history of AF. As highlighted by Bianco et al.,^7^ the emergence of AF after ablation of CTI-dependent AFL has great clinical relevance due to the high risk of thromboembolic events associated with AF, particularly stroke. The presence of AF is associated with 4-5 times greater risk of developing ischemic stroke. In a study including patients undergoing ablation of CTI-dependent AFL, the incidence of stroke over a mean follow-up of 40 months was four times greater than the general population.^8^ For this reason, considering the high incidence of AF in this population, discontinuation of oral anticoagulation may expose them to the risk of thromboembolic events and hence should be considered individually, considering the CHA2DS2-VASC score of the patient with FLA, just as with patients with AF.^2,9^

In addition, a significant number of patients remains symptomatic due to the emergence of AF following CTI-dependent AFL ablation. A second ablation procedure may then be necessary to control AF. Therefore, the question arises whether we should perform concomitant PVI in patients undergoing CTI-dependent AFL ablation, even before AF has ever occurred (10-12). Although AF ablation, by means of pulmonary vein isolation (PVI), is a more complex procedure, involving transseptal punctures, more extensive atrial ablation and use of three-dimensional mapping equipment, with higher costs and risks compared with CTI-dependent AFL ablation, an alternative strategy may be to treat both arrhythmias in a single ablation procedure, thus avoiding a second intervention.^7^

In this context, recent data suggest that prophylactic PVI can be an effective strategy for preventing new AF in patients undergoing CTI-dependent AFL ablation.^10-12^ The PREVENT AF I Study included 50 patients with CTI-dependent AFL without any prior history of AF, and randomized them in 1:1 fashion between CTI ablation alone versus CTI ablation plus cryoballoon PVI (CTI+PVI).^10^ New-onset AF occurred in 52% of CTI ablation alone versus 12% with CTI+PVI group over 1-year follow-up (p=0.003). Subsequently, Romanov et al.^11^ presented the extended (3-year) outcomes of the PREVENT AF I Study. There was a highly significant improvement in freedom from any atrial tachyarrhythmia in the CTI+PVI group compared with the CTI ablation only group (48% vs. 20%, P=0.01). Of note, there were no adverse events in the CTI+PVI group, but 2 strokes occurred in the CTI-only group during follow-up. In this study, a multivariate analysis identified male gender and age over 55 as factors that predicted atrial arrhythmias during follow-up. Additionally, the REDUCE AF study^12^ randomized 216 patients with lone AFL to CTI+PVI versus CTI ablation alone, and found a reduction in subsequent AF with prophylactic PVI, but at the cost of significantly longer procedure and fluoroscopy times. In *post hoc* analysis, all of the benefit was confined to those patients over 55 years of age, in agreement with the findings of Romanov et al.^11^

More recently, Gula et al.^13^ conducted a cost-effectiveness analysis comparing the strategy of combined prophylactic PVI plus CTI versus sequential approach with separate procedures, i.e., waiting for AF to occur before undergoing PVI. Making plausible projections on AF occurrence and PVI success rates, as well as risks and costs of the procedures, the authors found that the combined approach with prophylactic PVI conferred higher risk and higher cost than the sequential approach during follow-up. However, one should consider the limitations of this study, and perhaps a strategy of combined prophylactic PVI plus CTI would have more favorable risk/benefit ratio if applied more selectively to patients at highest risk for developing AF during follow-up.

In this context, as have been acknowledged by Bianco et al.,^7^ a significant limitation of their study was the limited size of the study population which may have prevented the identification of predictors for development of AF after CTI ablation. As recognized by the authors,^7^ if a risk profile for the occurrence of AF following CTI-dependent AFL ablation could be determined, a combined approach, including ablation of both arrhythmias, could be prophylactically indicated in patients at higher risk for developing AF.

In summary, the study of Bianco et al.^7^ provides further evidence that CTI-dependent AFL ablation is a safe and effective procedure, but solves just part of the clinical problem of the patient presenting with isolated AFL, since the occurrence of AF after CTI ablation is frequently observed. However, with regard to the issue highlighted by the authors,^7^ there is still insufficient evidence to recommend combined ablation for treatment of AFL aiming at preventing the occurrence of AF. Prospective studies with a larger number of patients and longer follow-up will are needed to assess the benefits of the simultaneous ablation.
